# Structural and Thermodynamic Characteristics of Amyloidogenic Intermediates of β-2-Microglobulin

**DOI:** 10.1038/srep13631

**Published:** 2015-09-08

**Authors:** Song-Ho Chong, Jooyeon Hong, Sulgi Lim, Sunhee Cho, Jinkeong Lee, Sihyun Ham

**Affiliations:** 1 Department of Chemistry, Sookmyung Women’s University, Cheongpa-ro-47-gil 100, Yongsan-ku, Seoul, 140-742, Korea

## Abstract

β-2-microglobulin (β2m) self-aggregates to form amyloid fibril in renal patients taking long-term dialysis treatment. Despite the extensive structural and mutation studies carried out so far, the molecular details on the factors that dictate amyloidogenic potential of β2m remain elusive. Here we report molecular dynamics simulations followed by the solvation thermodynamic analyses on the wild-type β2m and D76N, D59P, and W60C mutants at the native (N) and so-called aggregation-prone intermediate (I_T_) states, which are distinguished by the native *cis*- and non-native *trans*-Pro32 backbone conformations. Three major structural and thermodynamic characteristics of the I_T_-state relative to the N-state in β2m protein are detected that contribute to the increased amyloidogenic potential: (i) the disruption of the edge D-strand, (ii) the increased solvent-exposed hydrophobic interface, and (iii) the increased solvation free energy (less affinity toward solvent water). Mutation effects on these three factors are shown to exhibit a good correlation with the experimentally observed distinct amyloidogenic propensity of the D76N (+), D59P (+), and W60C (−) mutants (+/− for enhanced/decreased). Our analyses thus identify the structural and thermodynamic characteristics of the amyloidogenic intermediates, which will serve to uncover molecular mechanisms and driving forces in β2m amyloid fibril formation.

Amyloid fibril formation in the osteoarticular tissues resulting from the aggregation of β-2-microglobulin (β2m) is the hallmark of dialysis-related amyloidosis (DRA)^1^. With the full renal activity, β2m released from the major histocompatibility complex class I is filtered and degraded in the kidney to retain its low plasma concentration^2^. Upon renal failure, on the other hand, a 60-fold increase in the β2m concentration is observed in the serum^2^. The resulting high concentration has been implicated to be the cause of the fibrillogenesis associated with DRA. However, a sensible correlation has not been detected between the fibril load in the osteoarticular tissues and the β2m concentration in the serum^3^, implying that additional factors and/or events must be involved in initiating the β2m aggregation *in vivo*. Furthermore, the monomeric form of β2m is highly stable under physiological conditions and does not exhibit a tendency to aggregate even at elevated concentrations *in vitro*^4^,^5^.

It is now widely recognized that partial unfolding, also called misfolding, of monomeric species is a necessary step to initiate aggregation^6^. In this regard, a long-lived folding intermediate has been identified in the kinetics study of β2m folding that adopts a non-native *trans*-Pro32 backbone conformation^7^,^8^,^9^,^10^,^11^. This partially unfolded intermediate, termed as I_T_-state, has been recognized as a crucial amyloidogenic precursor since enhanced fibrillogenesis has been observed in variants of β2m in which the non-native *trans*-Pro32 isomer is stabilized^5^,^11^,^12^,^13^. However, it has also been reported that some mutants of β2m, which predominantly adopt a *trans* conformation at the proline residue 32, cannot form amyloid fibrils spontaneously^13^, indicating that the *trans* peptide conformation at this position alone is insufficient to endow amyloidogenic potential to β2m. It therefore remains to investigate in more details the changes in structural and possibly thermodynamic features induced by the *cis* to *trans* Pro32 backbone isomerization in order to unveil the characteristics of more direct relevance to the amyloidogenic propensity of β2m.

In this paper, we report *in silico* studies on the structural and thermodynamic features of the native (N) and intermediate (I_T_) states of the wild-type β2m and its D76N, D59P, and W60C mutants. Molecular dynamics (MD) simulations were conducted to analyze the atomic-level structural changes caused by the *cis*-to-*trans* Pro32 isomerization. Solvation free energy calculations using the molecular theory of solvation were also carried out to analyze thermodynamic consequences of the structural changes upon the Pro32 isomerization. Mutation effects were also studied to examine whether the structural and thermodynamic traits that we observe at the I_T_-state are indeed responsible for the amyloidogenic potential. The three mutants, D76N, D59P, and W60C, were chosen because of their distinct aggregation propensity^14^,^15^; the pathogenic familial mutant D76N and synthetic mutant D59P are known to exhibit more enhanced amyloidogenic propensity than the wild-type β2m, whereas the synthetic mutant W60C is less aggregation-prone. Thereby, we aim to identify the structural and thermodynamic characteristics of the amyloidogenic intermediates of β2m.

## Results

### Protein structures at the N-state are highly stable

**Figure 1:**
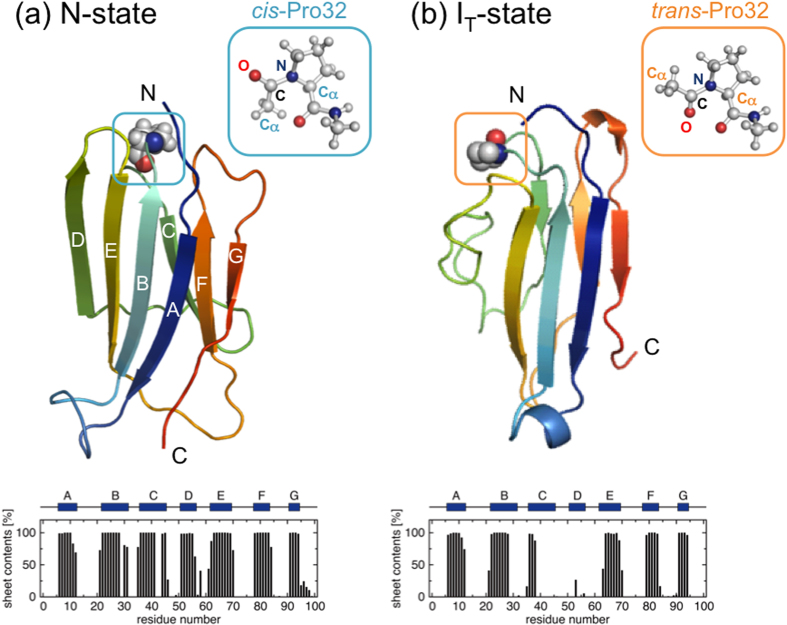
Representative wild-type structures at N-state (a) and I_T_-state (b) containing, respectively, the native *cis*- and a non-native *trans*-Pro32 residue. The average β-sheet contents versus amino acid residue are also displayed.

The wild-type β2m (99 residues) determined by X-ray (PDB entry 2YXF^16^), in which Pro32 adopts a native *cis*-conformation, shows an immunoglobulin fold comprising seven β-strands (termed as A to G) organized into two β-sheets (ABED and CFG) connected with a disulfide bridge (Cys25-Cys80) (see Fig. 1a). The X-ray structures of the D76N (PDB entry 4FXL^14^), D59P (3DHM^15^), and W60C (3DHJ^15^) mutants are quite similar to the wild-type structure. Indeed, Cα root-mean-square deviation (RMSD) values are within 0.8 Å between all of these X-ray structures.

**Figure 2:**
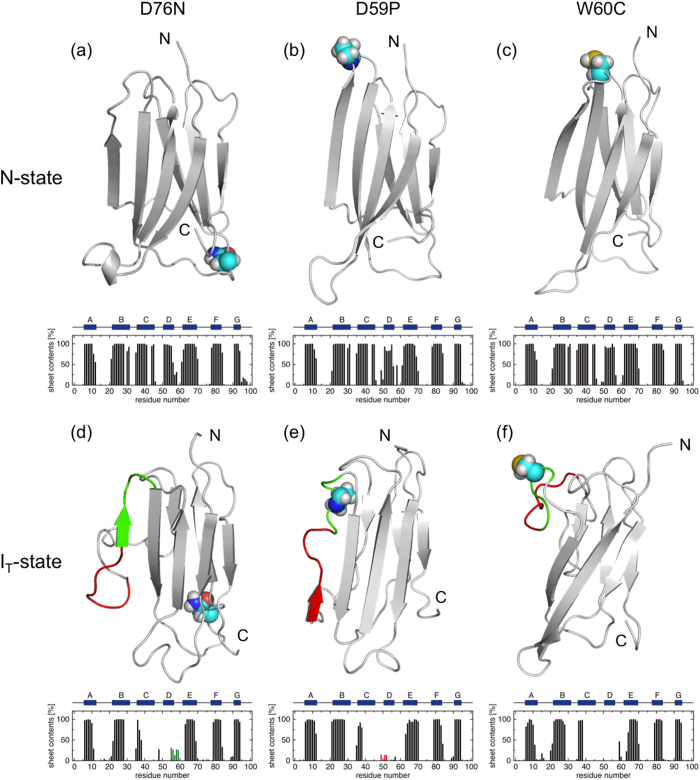
Representative N-state structures of D76N (a), D59P (b), and W60C (c) and I_T_-state structures of these proteins in this order (d–f). The mutation sites are represented with the space-filling model. The average β-sheet contents versus amino acid residue are also displayed. In (**d–f**), the D-strand and DE-loop regions are colored with red and green, respectively.

We performed the N-state simulations of the wild-type β2m and its D76N, D59P, and W60C mutants starting from the respective X-ray structures. The protein conformations during the N-state simulations were found to remain close to the X-ray structures (Table 1, Figs 1a and 2a–c): the average Cα RMSD values from the respective X-ray structures are within a few Å, the radii of gyration are nearly the same as those of the X-ray structures, the fraction of the native hydrophobic contacts are ~90%, and the β-sheets as found in the X-ray structures are well conserved. Thus, the native structures of the wild-type β2m as well as the D76N, D59P, and W60C mutants are highly stable at the simulated condition.

### Structural characteristics of the wild-type β2m at the I_T_-state

We carried out the I_T_-state simulations of the wild-type β2m, in which Pro32 takes a non-native *trans* conformation (Fig. 1b), to investigate structural changes induced by the Pro32 isomerization. The simulations were initiated from the partially unfolded structure obtained at a high temperature to enhance the otherwise slow *cis* to *trans* Pro32 isomerization (see Methods for details).

**Figure 3:**
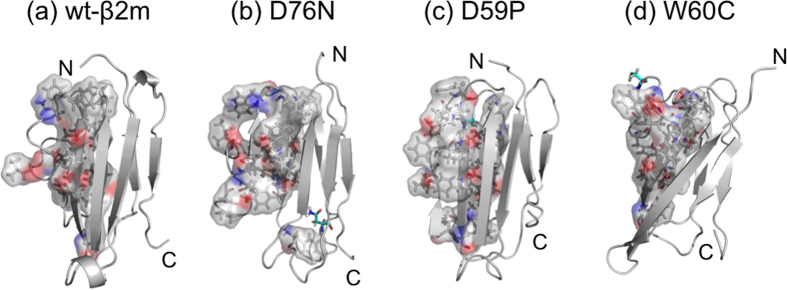
Surface representation of the hydrophobic residues located in the C-, D-, E-strands and CD- and DE-loop regions (residues 36 to 69) of the wild-type β2m (a), D76N (b), D59P (c), and W60C (d) in their representative I_T_-state conformations. The mutation sites are indicated with cyan stick representation.

The global structure and the overall topology of the wild-type β2m are well conserved between the N- and I_T_-states as can be inferred from the representative structures and the average β-sheet contents displayed in Fig. 1a,b, with the most notable exception being the unstructured edge D-strand at the I_T_-state. The disordered D-strand is accompanied by the disruption and solvent-exposure of the hydrophobic core residues surrounding this region (Fig. 3a). Indeed, the fraction of the native hydrophobic contacts decreases to ~50% (Table 1) and the solvent accessible surface area (SASA) of hydrophobic residues increases (Table 2) at the I_T_-state. However, the number of hydrophobic contacts (25.3) of the wild-type β2m at the I_T_-state is not decreased much as compared to the one (32.8) at the N-state (Table 1), indicating that a reorganized, or repacked, hydrophobic core is formed at I_T_-state.

### Mutation effects on the structural characteristics at the I_T_-state

The I_T_-state simulations for the three mutants, D76N, D59P, and W60C, were performed to investigate the mutation effects on the I_T_-state structural characteristics. The overall structural features are conserved between N- and I_T_-states also for these mutants as demonstrated in Fig. 2a–f. On the other hand, we find the increased β-sheet forming propensity around the edge D-strand region of the D76N and D59P mutants at the I_T_-state (Fig. 2d,e), whereas the D-strand remains unstructured in the W60C mutant (Fig. 2f).

The hydrophobic cores are also disrupted and reorganized in all the mutants at the I_T_-state compared to the N-state (Table 1). However, the degrees of the disruption and the resulting solvent exposure of the hydrophobic-core residues exhibit distinct behavior depending on the site of mutation. We find that the hydrophobic SASA at the I_T_-state is larger in the D76N and D59P mutants than in the wild-type β2m, whereas it is smaller in the W60C mutant (Table 2). This is exemplified in Fig. 3a–d showing the hydrophobic residues located in the C- to E-strand regions of the representative I_T_-state structures. It is observed that the solvent-exposure of the hydrophobic residues is substantial in the D76N and D59P mutants, whereas the solvent-exposed hydrophobic residues are rather compact in the W60C mutant.

### Solvation thermodynamic characteristics of the I_T_-state

While the structural features in the I_T_-state caused by the Pro32 isomerization presented so far are candidates of the protein intrinsic factors relevant to understanding amyloidogenic potential, it is also necessary to pay attention to environmental factors such as the role of solvent water. Indeed, two negatively charged β2m proteins (the total charge of the wild-type β2m at neutral pH is −2) would not approach each other if there were no water-mediated driving force to overcome the electrostatic repulsion. (This is true also for proteins in which positively and negatively charged residues are unevenly distributed on the protein surface; See Supplementary Discussion and Fig. S1 on this point.) The key quantity that quantifies the water-mediated interaction is the solvation free energy Δ*G*_solv_ measuring an overall affinity of a protein to water^17^. We computed Δ*G*_solv_ of the wild-type β2m both at the N- and I_T_-states by using the molecular theory of solvation (see Methods), and found that Δ*G*_solv_(I_T_) is larger than Δ*G*_solv_(N) by +47.9 kcal/mol. Since a larger Δ*G*_solv_ value is associated with a less affinity toward solvent water, this indicates that the I_T_-state protein conformations are more “hydrophobic” and possess a more tendency to self-associate in aqueous environments than the N-state conformations.

**Figure 4:**
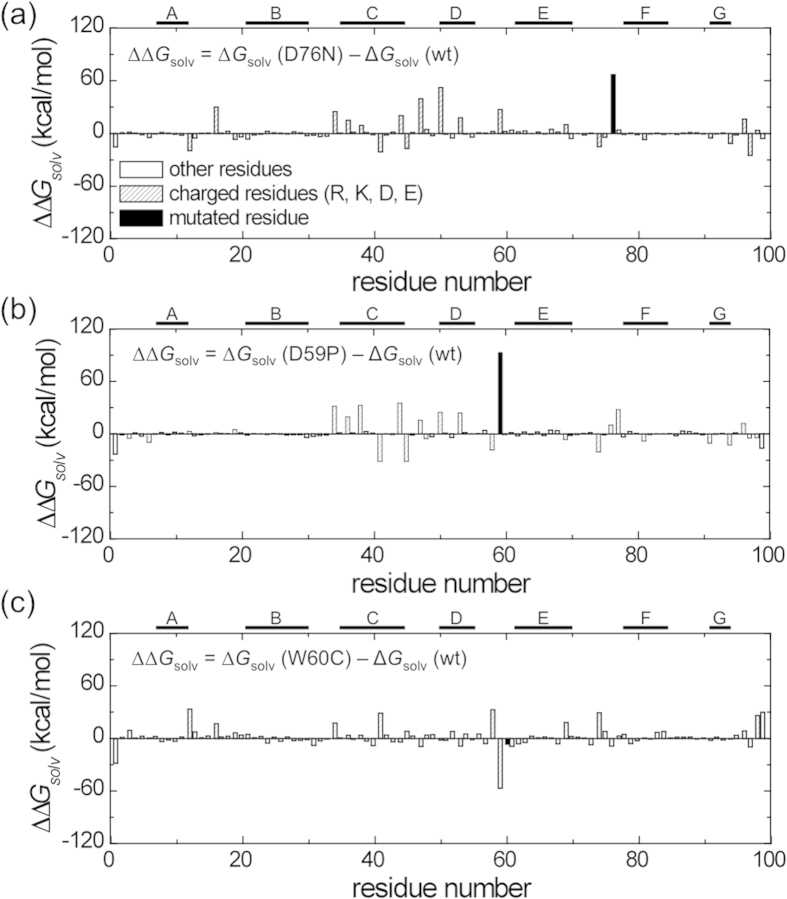
Contribution from each amino acid residue to the solvation free energy change, ΔΔ*G*_solv_ = Δ*G*_solv_(mutant) − Δ*G*_solv_(wild type), upon mutation at I_T_-state for D76N (a), D59P (b), and W60C (c).

Solvation free energies for the D76N, D59P, and W60C mutants at the I_T_-state were also computed to examine the mutation effects, and the results relative to the wild-type β2m at the I_T_-state, Δ*G*_solv_(mutant) − Δ*G*_solv_(wild type), are presented in Table 3. The solvation free energies for D76N and D59P mutants are found to be significantly larger than the one for the wild-type, whereas the one for W60C is lower. These results indicate that the water-induced attraction is stronger for the D76N and D59P mutants than the one for the wild type, while it is weaker for the W60C mutant. We also note that the mutation site is not the sole cause of the solvation free energy change upon mutation. Indeed, the residue-decomposed solvation free energy values illustrate the significant contributions from the amino acid residues other than the mutation site (Fig. 4). In particular, the C- through E-strand regions contribute to total solvation free energy changes by adapting the conformational changes upon mutation. It is noted that the large contributions from the charged residues reflect the fact that the electrostatic component dominates the change in solvation free energy upon mutation^18^,^19^.

## Discussion

Crucial to uncovering molecular mechanisms behind amyloid fibril formation is to elucidate factors that confer amyloidogenic potential to otherwise an inert soluble protein. Structural changes induced by the native *cis* to a non-native *trans* Pro32 isomerization has therefore been a subject of intense experimental investigations^12^,^20^,^21^,^22^,^23^,^24^. While detailed structural analysis of amyloidogenic intermediates has been difficult to investigate because of their transient nature, it has recently been demonstrated that the D-strand at I_T_-state exhibits less populated β-sheet conformation compared to N-state^23^ and that a repacking and exposure of the hydrophobic residues occur to accommodate the non-native *trans*-Pro32 conformation^12^,^20^. We find the disruption of the D-strand (Fig. 1) and the repacked (Table 1) as well as solvent-exposed (Table 2) hydrophobic residues at I_T_-state relative to N-state, and these structural features observed in our I_T_-state simulations are fully consistent with the experimental measurements.

We analyzed the D76N, D59P, and W60C mutants to examine whether the structural features observed in the simulated I_T_-state are relevant to experimentally observed amyloidogenic propensity. In this regard, the larger β-sheet forming propensity and the exposure of hydrophobic residues have been suggested as major factors dictating the protein aggregation propensity^25^,^26^. In our simulated I_T_-state structures, the enhanced β-sheet forming propensity was observed in the disordered D-strand region of the D76N and D59P mutants (Fig. 2d,e), while no such enhancement was detected in the W60C mutant (Fig. 2f). Furthermore, we find that D76N and D59P exhibit larger hydrophobic SASA than the wild-type β2m, while W60C shows smaller hydrophobic SASA (Table 2). These mutation-dependent structural features correlate quite well with an enhanced aggregation propensity of D76N^14^ and D59P^15^ and a reduced propensity of W60C^15^, indicating that these structural features are the characteristics associated with the amyloidogenic potential. Notably, the region where these structural changes are observed upon the Pro32 isomerization is the edge D-strand region whose potential role in assembling into amyloid fibrils has been proposed^27^,^28^,^29^.

Interaction with surrounding water of a protein also plays a critical role in dictating the protein aggregation propensity^30^. Indeed, it has been demonstrated for a substantial number of mutants that the change in protein aggregation propensity upon mutation has a significant correlation with the change in solvation free energy^30^. Furthermore, the decrease in solvation free energy has been shown to be the major driving force for proteins of the same charge to approach from a long distance to a short contact^31^,^32^. This indicates that a protein of a larger solvation free energy has a more tendency to self-associate. In this regard, we find at the I_T_-state that the D76N and D59P mutants exhibit a larger solvation free energy than the wild type, while the W60C mutant shows a smaller solvation free energy (Table 3). These trends are also in accord with the experimentally observed amyloidogenic propensity of these mutants. Such changes in the solvation free energy upon mutation are the results of structural changes induced by the Pro32 isomerization (Fig. 4). Thus, our structural and solvation thermodynamic analyses suggest that the β-sheet forming propensity of the disordered edge D-strand region and the solvent exposure of the hydrophobic residues, together with a concomitant solvation free energy change, are responsible for the amyloidogenic potential of the I_T_-state of β2m.

In summary, we report *de novo* computational studies for characterizing aggregation-prone intermediate (I_T_) state of the wild type and three mutants (D76N, D59P, and W60C) of β2m to investigate the molecular determinants of their distinct amyloidogenic potential. We focus on the changes in the structural and thermodynamic features caused by the native *cis* (N-state) to a non-native *trans* (I_T_-state) isomerization of Pro32, which has been recognized as a trigger to initiate the β2m aggregation. We find for the wild-type β2m that the edge D-strand becomes unstructured, the hydrophobic core is disrupted and solvent-exposed, and the solvation free energy is consequently increased upon the Pro32 *cis*-*trans* isomerization. These features are mutation-dependent, and through the comparison with the experimentally-measured aggregation propensity, it is suggested that (i) more enhanced β-sheet forming propensity of the disordered D-strand region, (ii) more solvent-exposed hydrophobic residues, and (iii) the larger value of the solvation free energy, are the structural and thermodynamic characteristics at the I_T_-state that impart more amyloidogenic potential. We believe that our work contributes to uncovering the structural motif and molecular mechanisms of β2m amyloid fibril formation.

## Methods

### MD simulations at N-state

Fully atomistic MD simulations with explicit water were performed at 330 K and 1 bar under neutral pH for the wild type as well as three D76N, D59P, and W60C mutants of β2m with the AMBER12 simulation suite^33^. The ff99SB force field^34^ was used for protein and the TIP3P model^35^ for water. The initial structures of the N-state simulations, in which Pro32 adopts a *cis*-conformation, were taken from X-ray: PDB entry 2YXF^16^ was used for the wild-type β2m, 4FXL^14^ for D76N, 3DHM^15^ for D59P, and 3DHJ^15^ for W60C. Each protein was solvated with ~10,000 water molecules. The control of temperature and pressure was done with Berendsen’s method^36^. The SHAKE algorithm^37^ was employed for bonds including hydrogen atoms, and a 2.0 fs time step was used.

We used the following common procedures for all MD simulations. After the energy minimization, a 20 ps equilibration simulation under constant volume was performed in which temperature was set to 330 K. (The choice of this temperature, which is higher than the room/body temperature, was intended to enhance the conformational changes at I_T_-state induced by the Pro32 isomerization, and for consistency, this temperature was also adopted for the N-state simulations.) We then carried out a 200 ps equilibration simulation with a constant pressure of 1 bar. Finally, production run at 330 K and 1 bar was conducted for 200 ns. Three independent production runs with differing initial random velocities were conducted for each system to compute statistical averages of various observables. No *cis* to *trans* Pro32 isomerization was observed during all N-state simulations.

### MD simulations at I_T_-state

We conducted partial unfolding simulations at a high temperature to obtain initial structures for the I_T_-state simulations in which Pro32 displays a non-native *trans* conformation. The high-temperature partial unfolding simulation was necessary since no atomic-level structure for the I_T_-state is available from experiments. It was also done because the *cis* to *trans* proline isomerization is one of the slowest protein conformational changes that is difficult to capture without an enhanced sampling method. There are other possible ways to obtain the I_T_-state from simulations, such as replica exchange simulations. In any case, high-temperature simulations or replicas are necessary to facilitate the Pro32 isomerization. At the same time, we consider it necessary to keep the high-temperature simulations short, in order to suppress high-temperature-induced conformational changes as much as possible. For these two reasons, we have chosen to simply perform short simulations at a high temperature, which are to be terminated as soon as the occurrence of the *cis*-to-*trans* Pro32 isomerization.

For the wild-type β2m, we carried out several short simulations at 600 K with a constant volume, starting from the N-state conformation equilibrated at 330 K and 1 bar. In one of the high-temperature trajectories, a *cis* to *trans* isomerization of Pro32 was observed at ~6 ns, and the protein structure just after the proline isomerization was taken as the starting structure of the I_T_-state simulations for the wild-type β2m. The initial structures of the I_T_-state simulations for the mutants (D76N, D59P, and W60C) were generated based on the one for the wild-type β2m by mutating the corresponding residues D76, D59, and W60 to N76, P59, and C60, respectively, using Swiss PDB Viewer^38^. Our method for generating the initial I_T_-state structures for the mutants is reasonable in light of the experimental observations that the *cis*-to-*trans* Pro32 isomerization is the trigger step to initiate the aggregation and that, before the occurrence of the Pro32 isomerization (i.e., in N-state), the structures of the D76N, D59P, and W60C mutants are quite similar to the wild-type structure (Cα RMSD values are within 0.8 Å between all of their X-ray structures). We then carried out three independent 200-ns I_T_-state production runs for each protein system. Pro32 maintained the *trans* conformation during the I_T_-state simulations in all cases.

To support our I_T_-state simulations initiated from the partial unfolding at a high temperature, we additionally performed 200 ns MD simulations for the wild-type β2m as well as its three mutants at the normal body temperature (310 K) and 1 bar starting from the protein structures containing a non-native *trans*-Pro32, which were obtained by locally changing the backbone torsions from the respective X-ray structures of *cis*-Pro32. We also observe from the supporting simulations that the most distinctive structural characteristic caused by the non-native *trans*-Pro32 is the disruption of the edge D-strand region, including the subtle mutation effect on the β-sheet forming propensity, which is consistent with our I_T_-state simulations (Supplementary Fig. S2). This indicates that the I_T_-state structural characteristics that we discuss in the main text are not due to the disruptive impact from the high temperature, but reflect genuine structural features caused by the *trans*-Pro32 conformation.

### Structural and solvation free energy analyses

Root-mean-square deviation of Cα atoms (Cα RMSD), radius of gyration (*R*_g_), secondary structure contents, number of hydrophobic contacts, and solvent accessible surface area (SASA) were computed to analyze the simulated protein structures. The DSSP^39^ was used to calculate the secondary-structure contents. A hydrophobic contact between a pair of hydrophobic residues is considered formed when the distance between the heavy side-chain atoms is less than 5.4 Å. Representative protein structures were obtained by performing the RMSD-based K-clustering analysis (3.5-Å cutoff).

We used the three-dimensional reference interaction site model (3D-RISM) theory^40^ to compute the solvation free energy Δ*G*_solv_ of the simulated protein conformations. This theory provides the equilibrium water distribution function around a given protein structure, from which Δ*G*_solv_ can be computed (see Supplementary Methods for details on the Δ*G*_solv_ calculations). For each protein system, we took 2,400 protein structures with a 250 ps interval from the three independent trajectories of 200 ns length to compute the average Δ*G*_solv_. An exact decomposition method is also available that divides Δ*G*_solv_ of a protein into contributions from the constituent amino acid residues^41^, which was used to analyze residue-specific contributions to the solvation free energy change upon mutation (see also Supplementary Methods for details).

Amyloid fibril formation involves a number of steps, including the initial partial unfolding of monomers to form the aggregation-prone (I_T_-state) structures, the oligomer formation from those monomers, and the final fibril growth through the attachment of monomers/oligomers. Overall, the structural and thermodynamic features of monomers at I_T_-state would play a crucial role. Therefore, although there is a huge difference between the timescale of our simulations (sub-microseconds) and that of the fibril formation measured experimentally (hours)^14^,^15^, our computational analyses revealing the I_T_-state characteristics will be relevant to uncovering the molecular determinants of the amyloidogenic potential.

## Additional Information

**How to cite this article**: Chong, S.-H. *et al.* Structural and Thermodynamic Characteristics of Amyloidogenic Intermediates of β-2-Microglobulin. *Sci. Rep.* **5**, 13631; doi: 10.1038/srep13631 (2015).

## Supplementary Material

Supplementary Information

## Figures and Tables

**Table 1:** Structural characteristics (average ± standard deviation).

	Cα RMSD(Å)^a^	*R*_g_ (Å)^b^	number ofhydrophobiccontacts	fraction of nativehydrophobic contacts(%)^c^
X-ray structure				
wild type	0	14.3	34	100
D76N	0	14.0	35	100
D59P	0	14.5	36	100
W60C	0	14.3	34	100
N-state				
wild type	2.4 ± 0.5	14.7 ± 0.1	32.8 ± 1.5	89.3 ± 3.6
D76N	2.6 ± 0.5	14.7 ± 0.1	32.8 ± 1.8	86.8 ± 3.9
D59P	2.1 ± 0.4	14.6 ± 0.1	33.4 ± 1.6	86.7 ± 3.3
W60C	2.0 ± 0.4	14.6 ± 0.1	33.2 ± 1.8	91.1 ± 3.3
I_T_-state				
wild type	4.9 ± 0.3	14.4 ± 0.2	25.3 ± 2.3	54.0 ± 3.9
D76N	5.8 ± 0.4	14.3 ± 0.2	26.7 ± 3.0	51.3 ± 3.8
D59P	6.2 ± 0.6	14.4 ± 0.2	25.2 ± 2.2	51.6 ± 3.9
W60C	6.2 ± 0.4	14.4 ± 0.2	23.0 ± 2.0	49.5 ± 3.4

^a^Cα RMSD relative to the respective X-ray structure.

^b^Radius of gyration.

^c^Fraction of the native hydrophobic contacts defined by the respective X-ray structure.

**Table 2:** Solvent accessible surface area (SASA) of hydrophobic side chains.

	hydrophobic SASA (Å^2^)^a^
N-state	
wild type	911.1 ± 58.3
D76N	882.3 ± 77.8
D59P	848.2 ± 59.5
W60C	770.3 ± 60.9
I_T_-state	
wild type	1037.2 ± 87.1
D76N	1057.7 ± 95.6
D59P	1142.3 ± 76.5
W60C	996.3 ± 104.7

^a^Average ± standard deviation.

**Table 3:** Solvation free energy change upon mutation at I_T_-state.

	ΔΔ*G*_solv_ (kcal/mol)^a^
D76N	+99.0 ± 26.2
D59P	+135.8 ± 9.9
W60C	−32.0 ± 13.1

^a^Difference in the solvation free energy, ΔΔ*G*_solv_ = Δ*G*_solv_(mutant) − Δ*G*_solv_(wild type), from the wild type ± standard error.
