# Supplementary material for: Structural and Thermodynamic Characteristics of Amyloidogenic Intermediates of β-2-Microglobulin
Source: Sci Rep. 2015 Sep 8;5:13631. doi: 10.1038/srep13631 (PMC4562173; doi:10.1038/srep13631)
Supplement: Supplementary Information [file srep13631-s1.pdf]

Supplementary Information for:

Structural and Thermodynamic Characteristics of Amyloidogenic Intermediates of β-2-Microglobulin

Song-Ho Chong, Jooyeon Hong, Sulgi Lim, Sunhee Cho, Jinkeong Lee, and Sihyun Ham*

Department of Chemistry, Sookmyung Women's University
Cheongpa-ro-47-gil 100, Yongsan-ku, Seoul, 140-742, Korea

Corresponding author:

*Sihyun Ham

Address	Department of Chemistry, Sookmyung Women's University<br>Cheongpa-ro 47-gil 100, Yongsan-ku, Seoul 140-742, Korea
Email	sihyun@sookmyung.ac.kr
Phone	+82-2-710-9410
Fax	+82-2-2077-7321

Supplementary Methods:

Solvation free energy calculations

To each of the simulated protein conformations, we applied the three-dimensional reference interaction site model (3D-RISM) theory[1] to compute the solvation free energy ΔG_{solv}. The 3D-RISM theory is an integral-equation theory based on statistical mechanics for obtaining the 3D distribution function $g_\gamma(\boldsymbol{r})$ of the water site γ (oxygen or hydrogen) at position $\boldsymbol{r}$ around a protein. For a solute-solvent system at infinite dilution, the 3D-RISM equation is given by

$$h_\gamma(\boldsymbol{r}) = \sum_{\gamma'} \int d\boldsymbol{r}' \chi_{\gamma\gamma'}(|\boldsymbol{r} - \boldsymbol{r}'|) c_{\gamma'}(\boldsymbol{r}') \quad ,$$

Here, $h_\gamma(\boldsymbol{r})$ and $c_\gamma(\boldsymbol{r})$ refer to the 3D total and direct correlation functions of the water site γ, and $\chi_{\gamma\gamma'}(r)$ denotes the site-site water susceptibility function, treated as an input to the theory, which can be obtained either from simulations or integral-equation calculations for pure water. This equation is to be supplemented by an approximate closure relation, another equation connecting $h_\gamma(\boldsymbol{r})$ and $c_\gamma(\boldsymbol{r})$ along with the solute-solvent interaction potential $u_\gamma(\boldsymbol{r})$, and in the present study we adopted the one developed by Kovalenko and Hirata,[1]

$$h_\gamma(\boldsymbol{r}) = \begin{cases} \exp\left[d_\gamma(\boldsymbol{r})\right] - 1 & \text{for} \quad d_\gamma(\boldsymbol{r}) \le 0 \\ d_\gamma(\boldsymbol{r}) & \text{for} \quad d_\gamma(\boldsymbol{r}) > 0 \end{cases}$$

in which $d_\gamma(\boldsymbol{r}) = -u_\gamma(\boldsymbol{r})/(k_B T) + h_\gamma(\boldsymbol{r}) - c_\gamma(\boldsymbol{r})$ with k_{B} denoting Boltzmann's constant. We used the same numerical procedure as described in Ref. 1 to self-consistently solve the above equations for $h_\gamma(\boldsymbol{r})$ and $c_\gamma(\boldsymbol{r})$. The water distribution function is then obtained via $g_\gamma(\boldsymbol{r}) = h_\gamma(\boldsymbol{r}) + 1$.

Solvation free energy ΔG_{solv} can be computed from Kirkwood's charging formula, $\Delta G_{\mathrm{solv}} = \rho \sum_{\gamma} \int_0^1 d\lambda \int d\boldsymbol{r}\, g_{\gamma}(\boldsymbol{r};\lambda)\, \partial u_{\gamma}(\boldsymbol{r};\lambda) / \partial\lambda$.[2] Here, λ is the charging parameter that gradually switches on the protein-water interaction from no interaction at $\lambda = 0$, $u_{\gamma}(\boldsymbol{r};\lambda = 0) = 0$, to the full interaction at $\lambda = 1$, $u_{\gamma}(\boldsymbol{r};\lambda = 1) = u_{\gamma}(\boldsymbol{r})$. $g_{\gamma}(\boldsymbol{r};\lambda)$ denotes the water distribution function corresponding to the parameter λ, and can be calculated from the 3D-RISM theory using the interaction potential $u_{\gamma}(\boldsymbol{r};\lambda)$.

Decomposition method of the solvation free energy

The interaction potential $u_{\gamma}(\boldsymbol{r})$ between the water site γ and the protein atoms consists of the pairwise additive Lennard-Jones (LJ) and electrostatic (elec) terms, $u_{\gamma}(\boldsymbol{r}) = \sum_i \left[u_{\gamma i}^{\mathrm{LJ}}(|\boldsymbol{r} - \boldsymbol{r}_i|) + u_{\gamma i}^{\mathrm{elec}}(|\boldsymbol{r} - \boldsymbol{r}_i|) \right]$, centered on the protein atom i of position $\boldsymbol{r}_i$. Substituting this into the Kirkwood charging formula yields an exact partitioning of the solvation free energy into the LJ and electrostatic terms, which can further be decomposed into contribution from each protein atom i,[2]

$$\Delta G_{\mathrm{solv}} = \Delta G_{\mathrm{solv}}^{\mathrm{LJ}} + \Delta G_{\mathrm{solv}}^{\mathrm{elec}} = \sum_i \left(\Delta G_i^{\mathrm{LJ}} + \Delta G_i^{\mathrm{elec}} \right)$$

Each residue contribution to the solvation free energy shown in Fig. 4 of the main text was obtained based on this decomposition formula.

Supplementary Discussion:

Protein aggregation/protein-protein binding is a complex process and many factors come into play. The aggregation propensity/binding affinity is affected, e.g., by the electrostatic interaction at the protein-protein interface. However, the strength of the electrostatic interaction can be nontrivial since positively and negatively charged residues in a protein are typically unevenly distributed on the protein surface.

Our statement in the main text – two negatively charged β2m proteins would not approach each other if there were no water-mediated driving force to overcome the electrostatic repulsion – comes from our previous simulation work on the spontaneous dimerization of the 42-residue amyloid-beta (Aβ42) proteins.[3] We found that, *after two monomers start to make atomic contacts*, the electrostatic and van der Waals interactions at the protein-protein interface play a central role. However, *before two monomers make atomic contacts, the inter-monomer interaction is essentially repulsive*, in spite of the fact that the positively and negatively charged residues are unevenly distributed on the Aβ42 surface. (Aβ42 monomer has a –3 net charge comprising 6 negatively charged residues and 3 positively charged residues). This is demonstrated in Supplementary Fig. S1: whereas the data points are scattered reflecting the heterogeneous charge distribution of Aβ42 (i.e., the inter-monomer interaction depends not only on the center-of-mass distance but also on the relative orientation), it is seen that the inter-monomer interaction is essentially Coulomb repulsion while two Aβ42 proteins approach from a large separation (~80 Å) to a contact distance (~30 Å). Although we cannot draw a corresponding figure for β2m since we did not conduct the dimerization simulation, it is expected that this is a general feature that applies when two proteins approach each other from a large separation to a contact distance.

Supplementary Figures:

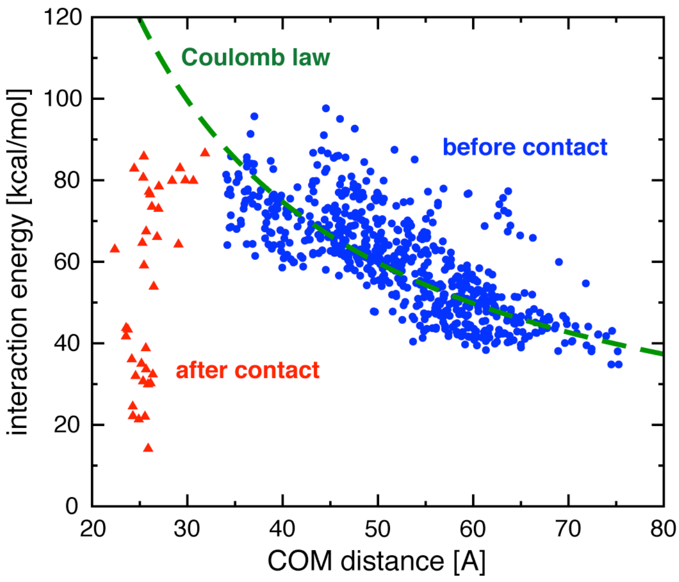

Figure S1. Inter-monomer interaction energy plotted as a function of the center-of-mass (COM) distance taken from the 0 to 35 ns time regime of the spontaneous Aβ42 dimerization simulation.[3] Data points referring to the time regimes before and after two Aβ42 monomers make atomic contacts are distinguished by blue and red colors, respectively. Green dashed line denotes the Coulomb law, Q^2/R, with Q being the total charge (–3) of an Aβ42 monomer and R the COM distance between two monomers.

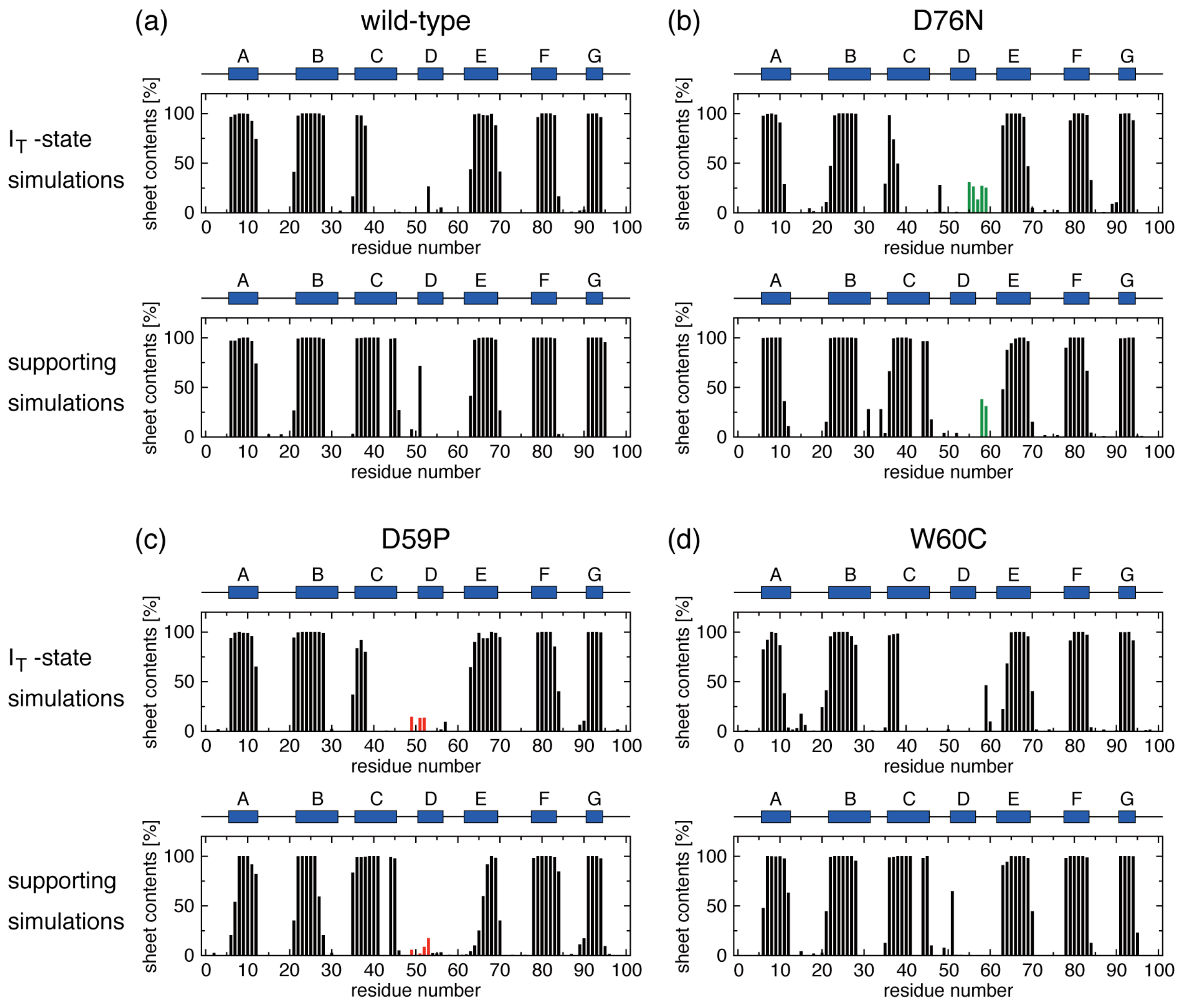

Figure S2. Average β-sheet contents versus amino acid residue for the wild-type β2m (a), D76N (b), D59P (c), and W60C (d). The upper panels refer to the results from the I_T-state simulations discussed in the main text, and are taken from Figs. 1b and 2d–2f for the respective systems. The lower panels display the results from the additional 200 ns supporting simulations performed at 310 K and 1 bar starting from the protein structures containing a non-native *trans*-Pro32, which were obtained by locally changing the backbone torsions from the respective X-ray structures of *cis*-Pro32 (see Methods); the results shown were obtained by averaging over the last 100 ns part of the trajectories.

References

1. Imai, T., Harano, Y., Kinoshita, M., Kovalenko, A. & Hirata, F. A theoretical analysis on hydration thermodynamics of proteins. *J. Chem. Phys*. **125**, 024911 (2006).
2. Chong, S.-H. & Ham, S. Atomic decomposition of the protein solvation free energy and its application to amyloid-beta protein in water. *J. Chem. Phys*. **135**, 034506 (2011).
3. Chong, S.-H. & Ham, S. Impact of chemical heterogeneity on protein self-assembly in water. *Proc. Natl. Acad. Sci. U.S.A*. **109**, 7636–7641 (2012).
